# Design and Evaluation of a Cloud Computing System for Real-Time Measurements in Polarization-Independent Long-Range DAS Based on Coherent Detection

**DOI:** 10.3390/s24248194

**Published:** 2024-12-22

**Authors:** Abdusomad Nur, Almaz Demise, Yonas Muanenda

**Affiliations:** 1 Addis Ababa Institute of Technology, Addis Ababa University, King George VI St, Addis Ababa 1000, Ethiopia; 2 Institute of Mechanical Intelligence, Scuola Superiore Sant’Anna, Via G. Moruzzi 1, 56124 Pisa, Italy; almazassire.demise@santannapisa.it (A.D.); y.muanenda@sssup.it (Y.M.)

**Keywords:** polarization-independent detection, distributed acoustic sensing, cloud computing, CloudSim

## Abstract

CloudSim is a versatile simulation framework for modeling cloud infrastructure components that supports customizable and extensible application provisioning strategies, allowing for the simulation of cloud services. On the other hand, Distributed Acoustic Sensing (DAS) is a ubiquitous technique used for measuring vibrations over an extended region. Data handling in DAS remains an open issue, as many applications need continuous monitoring of a volume of samples whose storage and processing in real time require high-capacity memory and computing resources. We employ the CloudSim tool to design and evaluate a cloud computing scheme for long-range, polarization-independent DAS using coherent detection of Rayleigh backscattering signals and uncover valuable insights on the evolution of the processing times for a diverse range of Virtual Machine (VM) capacities as well as sizes of blocks of processed data. Our analysis demonstrates that the choice of VM significantly impacts computational times in real-time measurements in long-range DAS and that achieving polarization independence introduces minimal processing overheads in the system. Additionally, the increase in the block size of processed samples per cycle results in diminishing increments in overall processing times per batch of new samples added, demonstrating the scalability of cloud computing schemes in long-range DAS and its capability to manage larger datasets efficiently.

## 1. Introduction

Optical fiber sensors constitute a key element of intelligent monitoring systems as they are adaptable, are resistant to harsh environmental conditions, and can be easily integrated into existing fiber-optic infrastructures. Among others, Distributed Acoustic Sensing (DAS) involves the use of an optical fiber to detect and measure acoustic signals over a long distance [1,2]. By transmitting coherent signals through the fiber and analyzing the coherent Rayleigh backscattered light and its temporal change at each point due to acoustic disturbances, DAS systems can transform fiber-optic cables into a continuous, high-resolution array of microphones [3,4]. This technology enables the monitoring of extensive areas of long infrastructure with a single fiber [5], making it particularly useful for applications in a number of areas include smart buildings and the transportation and energy industries.

DAS has various applications ranging from offshore leakage detection in oil and gas wells and pipelines to perimeter security [6], as well as monitoring the structural safety and integrity of critical infrastructure including railways and highways [7] and long-haul power transmission lines. A common scheme for DAS is coherent OTDR, also known as phase-sensitive OTDR (φ-OTDR), wherein the coherent Rayleigh backscattering from pulses of light from a narrow linewidth laser are observed with regard to the change in intensity and phase caused by local disturbances. Monitoring the evolution of the change in amplitude and phase of the backscattering along the fiber provides information on the distributed temperature or the vibrations induced by events which leave specific signatures. For continuous monitoring, especially at long distances, multiple cycles of traces, each with hundreds of thousands of samples, need to be acquired and stored for further processing to extract information on the magnitude, location, and frequency of disturbances [8].

In φ-OTDR, the coherent Rayleigh backscattering from each point along the fiber consists of a speckle pattern determined by the randomly varying birefringence along the fiber. In coherent detection in DAS via beating of the backscattering with a local oscillator having a certain polarization, the received signal is inevitably affected by polarization mismatch, ultimately resulting in polarization fading, which is detrimental to the measurement. This might necessitate the use of expensive polarization-maintaining fibers as a sensor. However, the use of polarization diversity mitigates this problem, thereby enabling accurate measurement of vibrations regardless of the type of fiber used and the state of polarization of the backscattered light from any position along the fiber [8].

### 1.1. Interrogation Schemes and Data Handling in DAS

**Figure 1:**
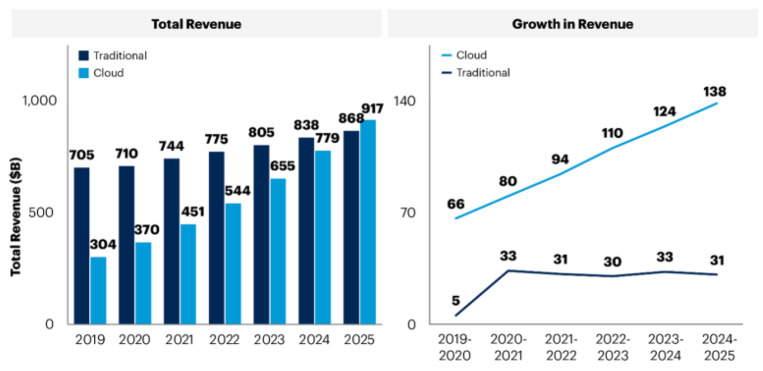
Trend of total revenue and growth revenue of enterprise IT spending, showing the trends in the use of cloud and traditional systems [9].

The use of cloud systems in enterprise IT solutions for fiber optics has been showing steady growth and continues to constitute a significant part of the total revenue, which is expected to be USD 1.3 billion in 2025, as per a recent survey [9]. As shown in Figure 1, not only the share of the total revenue but also the growth in revenue of cloud computing have been steadily growing, in clear contrast to traditional systems, which have shown stagnation.

While processing these data with conventional algorithms for real-time event extraction in itself remains difficult, in response to the growing demand for intelligent solutions, future monitoring systems require more valuable postprocessing on large datasets, which will be difficult to handle using standalone computing systems. There are studies which tackle the real-time processing of large amounts of data representing distance, depth, and wave velocity density obtained from DAS arrays in micro-seismic monitoring using deep learning using convolutional neural networks [10].

The high volume and velocity of DAS data necessitate big data tools, including cloud-based storage and analytics for scalable, real-time analysis. Data science and cloud solutions are expected to enhance DAS applications in environmental monitoring and IoT, especially within smart cities. As DAS sensors generate diverse data, cloud IoT applications and big data tools facilitate effective data management for large-scale, multi-parameter monitoring systems [11,12]. Considering the additional postprocessing needed to extract relevant information from raw traces, designing and evaluating the use of a cloud-based signal processing system capable of handling algorithms of varying degrees of complexity in the high volume of data involved in Distributed Acoustic Sensing is highly desirable.

Research on DAS has focused on improving the cost-effectiveness and precision of interrogation schemes, exploring its applications in security, and integrating it with advanced technologies [13,14]. The Distributed Fiber-Optic Intrusion Sensor (DFOIS), based on φ-OTDR, detects and locates intruders by sensing phase changes due to pressure on buried fibers [15]. Enhanced fiber designs, with better signal-to-noise ratios, allow accurate, rapid monitoring, beneficial for seismic activity detection, disaster monitoring, and oil exploration [13]. Machine learning and data science are crucial for processing DAS’s vast data, providing actionable insights for applications in industrial monitoring, smart cities, and infrastructure integrity [11]. DAS can also be used to detect intrusions by monitoring acoustic signals along fiber routes, boosting situational awareness and response [12].

More recently, the increase in the number and complexity of devices in the IoT architecture has necessitated edge and cloud computing [16]. Cloud simulators enable analysis of multiple parameters for a large number of devices and offer application interfaces for visualization [17], including in the placement and optimization of edge server computing [18].

Among others, a pioneering deployment of a fiber-optic DAS system for permanent flow monitoring in a tight gas well in Northern British Columbia was explored [19]. The system was installed with three main goals: continuous wellbore flow monitoring, real-time data access for team collaboration, and refining DAS for future oil and gas applications. This DAS solution enhances well and reservoir surveillance, often constrained by risks, costs, and logistical issues in traditional monitoring. With constant access to high-resolution flow data, DAS enables high-frequency monitoring, ideal for unconventional reservoir projects. Results confirm DAS’s capability to record real-time flow data accessible via a secure web interface, though challenges remain with large data volumes, data transfer, and system durability.

### 1.2. Advanced Signal Processing in DAS and Big Data Systems

A number of signal processing schemes have been used for event detection in DAS [2]. Conventional methods often focus on basic event detection but lack robust classification capabilities and tend to produce high false alarm rates [20]. To address this, machine learning (ML) approaches for event detection using DAS data are used. For instance, ML significantly enhances optical fiber sensor capabilities as it can be used to improve signal demodulation, enhance discrete and distributed sensor accuracy, and advance optical fiber speckle pattern processing. There are also studies where machine learning was used in optical fiber sensors to address issues with phase demodulation algorithms [21]. Furthermore, the use of convolutional neural networks able to classify events with methods employing the phase and intensity stacking in a φ-OTDR based on coherent detection has been proposed and demonstrated [22]. The use of the algorithm on a dataset for measuring the phase and intensity changes was shown to yield classification accuracy of 88.2%.

The integration of machine learning with DAS offers a transformative approach to pipeline monitoring, enhancing the ability to detect and classify potential threats to pipeline integrity. Machine learning enhances the utility of DAS by automating the recognition of patterns in the captured vibration data [23]. Features are then extracted from the processed signals in either the time domain (e.g., energy distribution, correlation), frequency domain (e.g., Fast Fourier Transform (FFT), spectral coefficients), or time–frequency domain (e.g., wavelet transforms). Among these, frequency-domain features are particularly effective due to their physical relevance and ability to distinguish between event types based on spectral characteristics [2], hence the need to focus on FFT signal processing for event extraction.

Furthermore, the integration of machine learning into DAS systems, referred to as DAS and Pattern Recognition Systems (PRS), not only reduces false alarms but also provides richer contextual information about the detected events. These systems process raw vibration signals into usable data through feature extraction, followed by classification to assign the signals to specific categories, such as threat or non-threat. A typical DAS and PRS framework comprises acquisition equipment to capture signals, a feature extraction module to generate feature vectors, and machine learning models to perform classification [23]. Pattern recognition in DAS has evolved with the integration of advanced signal processing and machine learning techniques. The combination of intensity and phase data, deep learning models, and data augmentation methods has significantly enhanced the capability of DAS systems to accurately classify events in real-time scenarios. These advancements ensure the practical deployment of DAS in complex environments, making it a robust solution for diverse monitoring applications [22].

Hence, it is evident that it is important to have scalable means to implement signal and data processing algorithm schemes in DAS with cloud computing systems for not only conventional processing systems but also new ones employing machine learning and pattern recognition for event extraction, identification, and classification.

The integration of cloud-based big data platforms into upstream oil and gas (O&G) operations to enhance productivity, safety, and cost efficiency was also addressed [24]. The study addresses industry challenges posed by rapid data growth from fiber-optic sensing, highlighting issues like data overload, incompatible systems, and secure data needs. To tackle this, a scalable, secure cloud-based platform for real-time downhole data processing was proposed using Apache Kafka for ingestion, Apache Spark for processing, and Apache Cassandra for storage. The cloud approach saves costs by reducing hardware and IT needs, offering reliability and scalability, and helping O&G operators focus on core objectives. The contribution shows that a cloud-based architecture enhances real-time data processing, supporting data-driven decision-making, profitability, and competitiveness in digital oilfield operations.

While different aspects of DAS have been studied in detail, there are limited investigations of tools and approaches for rendering long-range distributed sensors suitable for real-time monitoring [25]. This is, to the best of our knowledge, the study of a cloud simulation tool for modeling the signal processing in sample distributed fiber sensing systems. One key step forward in this direction requires quantifying the type, number, and specifications of resources required for a scenario of real-time monitoring with dynamic distributed sensing. Specifically, the intermediate data handling and signal processing in a DAS system involves acquisition of multiple traces and subsequent processing with spectral computations and phase demodulation techniques. Given that, often, knowing the exact storage and processing resources for a given system requires tests on real systems, which are costly, it is convenient to use tools which simulate the cloud architecture and allow prediction of expected performances in simple, readily available machines.

One such tool is CloudSim, which has been used in many design scenarios spanning a number of applications. The tool has been widely used by researchers and engineers with various approaches, including ones for simple processing [26], and adaptations for simulation of distributed functions as a service (FaaS) [27]. The various scheduling algorithms in cloud environments have been captured by the simulation tool and shown to serve as effective design tools to determine cloud solutions for multiple scenarios. Among others, CloudSim has been used to simulate computations in smart grids by studying parameters such as the number and bandwidth of VMs as well as the RAM and cloudlet length [28].

In this contribution, we design and evaluate a cloud computing scheme using the CloudSim simulation tool, which takes in data in the format of raw traces acquired from a DAS system for polarization-independent detection of distributed vibrations. The algorithms for extracting the phase by combining the in-phase and quadrature components of the coherent Rayleigh backscattering signal in the fast and slow polarization axes are implemented in the CloudSim tool. A comparison of the performances in terms of reduced processing times for different specifications of virtual machines with varying RAM size and processing power has been performed for one or more of the sequential algorithms needed to extract the response of the fiber from raw data. Our analysis informs implementations of a cloud computing system for real-time signal processing in a distributed dynamic sensing system.

This paper is organized as follows: The theory of signal processing with polarization diversity coherent detection of coherent Rayleigh backscattering is presented in Section 2.1, followed by a brief description of CloudSim in Section 2.2. Then, in Section 3, we present the experimental setup of the proposed interrogation system, while Section 4 outlines the design of the signal processing scheme in CloudSim. Plots of experimental and analysis results and respective descriptions appear in Section 5, followed by additional discussions and conclusions in Section 6.

## 2. Theory

### 2.1. Operating Principle of Polarization Diversity Hybrid

Coherent optical systems permit a low signal-to-noise ratio and compensate for several types of propagation impairments while preserving phase information of the optical signal. When a pulse of light from a highly coherent laser source is delivered to the sensing fiber, the phase of the backscattered Rayleigh light conveys the vibration information [29,30].

**Figure 2:**
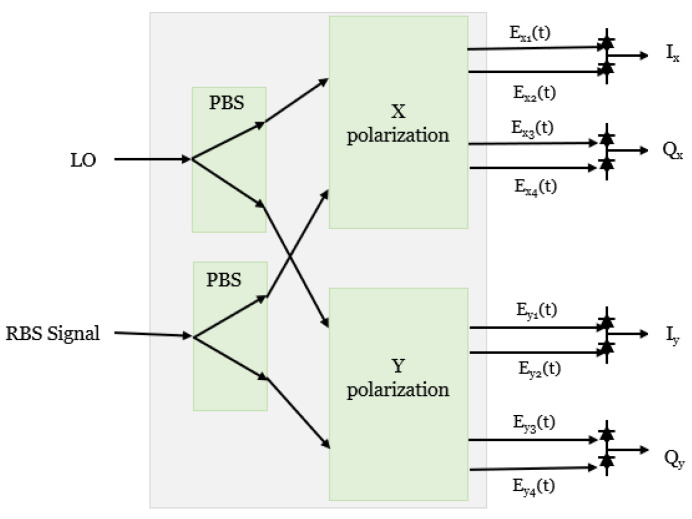
Configuration of the polarization diversity hybrid with a balanced photodiode. PBS: polarizing beam splitter.

Coherent systems operating with a single polarization typically include a receiver in which the received signal is mixed with a local oscillator (LO) operating at a frequency close to the former. Then, the LO and Rayleigh backscattering (RBS) signal are fed to an optical hybrid, followed by a pair of balanced photodetectors (PBDs) for each of the in-phase and quadrature components of the beat signal and subsequently digitized with an analog-to-digital converter (ADC) which feeds a Digital Signal Processing (DSP) system to extract the change in amplitude and phases induced by perturbations along the fiber [31]. The balanced detection suppresses the common-mode noise between the signal and the LO. However, due to the fiber’s randomly varying birefringence, the backscattering signal’s polarization is not always aligned with that of the LO. To solve this problem, a polarization diversity receiver is employed in a coherent receiver. Figure 2 shows the configuration of the generic polarization diversity coherent receiver, wherein two optical hybrids are combined in a polarization diversity configuration. First, the incoming signal with an arbitrary state of polarization (SOP) is separated into two linear polarization components with a polarization beam splitter (PBS).

In a φ-OTDR-based DAS system, the detected signal is the probe pulse’s coherent RBS. Assume the complex electric field of RBS light can be described as

(1) $$E_{s}\left(t\right)=A_{s}\left(t\right)exp^{j(\omega_{s}t+\theta_{s}\left(t\right)+\theta \left(t\right))}$$

where $A_{s}$(*t*), $\omega_{s}$(*t*), and $\theta_{s}$ (*t*) are the complex amplitude, the angular frequency, and the initial phase of the signal, respectively, and θ(*t*) is the phase change induced by the refractive index variation along the fiber. Similarly, the complex electric field of the LO used as a reference at the receiver can be written as

(2) $$E_{L}\left(t\right)=A_{L}exp^{j(\omega_{L}t+\theta_{L}\left(t\right)+\theta \left(t\right)),}$$

where $A_{L}$, $\omega_{L}$, and $\theta_{L}$ (*t*) are the complex amplitude, the angular frequency, and the phase of the local oscillator, respectively. $A_{L}$ is a constant given that the local oscillator has a continuous wave. The incoming signal has an arbitrary SOP and LO, which are separated into two linear polarization components (*x* and *y*) with a PBS. Each polarization is then given to the 90° optical hybrid. Output signals from the PBS are given by

(3) $$E_{sx}\left(t\right)=A_{sx}\left(t\right)exp^{j(\omega_{s}t+\theta \left(t\right)+\theta \left(t\right))}$$

(4) $$E_{sy}\left(t\right)=A_{sy}\left(t\right)exp^{j(\omega_{s}t+\theta_{s}\left(t\right)+\theta \left(t\right))}$$

(5) $$E_{Lx}\left(t\right)=A_{Lx}exp^{j(\omega_{L}t+\theta_{L}\left(t\right))}$$

(6) $$E_{Ly}\left(t\right)=A_{Ly}exp^{j(\omega_{L}t+\theta_{L}\left(t\right))},$$

where $E_{sx}$(*t*), $E_{sy}$ (*t*), $A_{sx}$ (*t*), and $A_{sy}$ (*t*) are complex electric fields and amplitudes of the transmitted signal in the *x* and *y* polarization, respectively.

Inside each polarization’s 90° hybrid, the LO power is split into two branches equally, and one of these arms is phase-shifted by 90°. Similarly, the RBS signal is split equally into two branches. The output signals of the *x* polarization from a 90° hybrid will be 1/2$E_{sx}$(*t*), 1/2$A_{Lx}$, 1/2$A_{jLx}$. The same thing happens in the *y* polarization, and the coefficient of ½ comes from the equal splitting of the signal.

Each branch of the LO output is coupled with the respective branch of the RBS signal with a 3 dB optical coupler that adds a 180° phase shift to either the signal field or the LO field. We can obtain four outputs $E_{x1}$ (*t*), $E_{x2}$ (*t*), $E_{x3}$ (*t*), and $E_{x4}$ (*t*) for the *x* polarization’s hybrid, as shown in Figure 2.

For each polarization state, we can obtain four electric field outputs that are incident on the upper and lower photodiodes as shown from Equation (7) to Equation (14). For the *x* polarization’s 90° hybrid, we can obtain the following outputs [32]:

(7) $$E_{x1}\left(t\right)=\frac{1}{2\sqrt{2}}\left(E_{sx}\left(t\right)+E_{Lx}\left(t\right)\right)$$

(8) $$E_{x2}\left(t\right)=\frac{1}{2\sqrt{2}}\left(E_{sx}\left(t\right)-E_{Lx}\left(t\right)\right)$$

(9) $$E_{x3}\left(t\right)=\frac{1}{2\sqrt{2}}\left(E_{sx}\left(t\right)+jE_{Lx}\left(t\right)\right)$$

(10) $$E_{x4}\left(t\right)=\frac{1}{2\sqrt{2}}\left(E_{sx}\left(t\right)-jE_{Lx}\left(t\right)\right)$$

Note that in Equations (9) and (10), j refers to the shifting of one arm of the LO by 90 degrees inside the *x* polarization hybrid.

We can similarly form the *y* polarization’s 90° hybrid:

(11) $$E_{y1}\left(t\right)=\frac{1}{2\sqrt{2}}\left(E_{sy}\left(t\right)+E_{Ly}\left(t\right)\right)$$

(12) $$E_{y2}\left(t\right)=\frac{1}{2\sqrt{2}}\left(E_{sy}\left(t\right)-E_{Ly}\left(t\right)\right)$$

(13) $$E_{y3}\left(t\right)=\frac{1}{2\sqrt{2}}\left(E_{sy}\left(t\right)+jE_{Ly}\left(t\right)\right)$$

(14) $$E_{y4}\left(t\right)=\frac{1}{2\sqrt{2}}\left(E_{sy}\left(t\right)-jE_{Ly}\left(t\right)\right)$$

The current output of each photodetector is proportional to the square of the magnitude of the total incident electric flux [33]. The incident wave on the first photodetector is $E_{x1}\left(t\right)$ which, using Equation (7), results in

(15) $$I_{x1}\left(t\right)=\frac{R}{8}[{\left(A_{sx}\left(t\right)\right)}^{2}+{\left(A_{Lx}\right)}^{2}+2A_{sx}\left(t\right)A_{Lx}cos(\left(\omega_{s}-\omega_{L}\right)t+\left(\theta_{s}\left(t\right)-\theta_{L}\left(t\right)\right)+\theta \left(t\right))]$$

where *R* is the responsivity of photodetectors. Using Equation (8) for the second photodetector, we obtain

(16) $$I_{x2}\left(t\right)=\frac{R}{8}[{\left(A_{sx}\left(t\right)\right)}^{2}+{\left(A_{Lx}\right)}^{2}-2A_{sx}\left(t\right)A_{Lx}cos(\left(\omega_{s}-\omega_{L}\right)t+\left(\theta_{s}\left(t\right)-\theta_{L}\left(t\right)\right)+\theta \left(t\right))]$$

(17) $$I_{x}\left(t\right)=I_{x1}\left(t\right)-I_{x2}\left(t\right)=\frac{R}{2}A_{sx}\left(t\right)A_{Lx}cos(\left(\omega_{s}-\omega_{L}\right)t+\left(\theta_{s}\left(t\right)-\theta_{L}\left(t\right)\right)+\theta \left(t\right))$$

In the quadrature output component of the photodiode, one arm of the LO is shifted by 90°, and using Equation (9) for the third photodiode, we obtain [31]

(18) $$Q_{x1}\left(t\right)=\frac{R}{8}[{\left(A_{sx}\left(t\right)\right)}^{2}+{\left(A_{Lx}\right)}^{2}+2A_{sx}\left(t\right)A_{Lx}cos(\left(\omega_{s}-\omega_{L}\right)t+\left(\theta_{s}\left(t\right)-\theta_{L}\left(t\right)\right)+\theta \left(t\right)+\frac{\pi }{2})]$$

Equation (18) is simplified as

(19) $$Q_{x1}\left(t\right)=\frac{R}{8}[{\left(A_{sx}\left(t\right)\right)}^{2}+{\left(A_{Lx}\right)}^{2}+2A_{sx}\left(t\right)A_{Lx}sin(\left(\omega_{s}-\omega_{L}\right)t+\left(\theta_{s}\left(t\right)-\theta_{L}\left(t\right)\right)+\theta \left(t\right))]$$

Using Equation (10) for the last photodiode of the *x* polarization gives

(20) $$Q_{x2}\left(t\right)=\frac{R}{8}[{\left(A_{sx}\left(t\right)\right)}^{2}+{\left(A_{Lx}\right)}^{2}-2A_{sx}\left(t\right)A_{Lx}sin(\left(\omega_{s}-\omega_{L}\right)t+\left(\theta_{s}\left(t\right)-\theta_{L}\left(t\right)\right)+\theta \left(t\right))]$$

The balanced detector output of the quadrature component is then given by

(21) $$Q_{x}\left(t\right)=\frac{R}{2}A_{sx}\left(t\right)A_{Lx}sin(\left(\omega_{s}-\omega_{L}\right)t+\left(\theta_{s}\left(t\right)-\theta_{L}\left(t\right)\right)+\theta \left(t\right))$$

Similarly, the corresponding values of in-phase and quadrature components of the *y* polarization are given by

(22) $$I_{y}\left(t\right)=\frac{R}{2}A_{sy}\left(t\right)A_{Ly}cos(\left(\omega_{s}-\omega_{L}\right)t+\left(\theta_{s}\left(t\right)-\theta_{L}\left(t\right)\right)+\theta \left(t\right))$$

(23) $$Q_{y}\left(t\right)=\frac{R}{2}A_{sy}\left(t\right)A_{Ly}sin(\left(\omega_{s}-\omega_{L}\right)t+\left(\theta_{s}\left(t\right)-\theta_{L}\left(t\right)\right)+\theta \left(t\right))$$

In this experiment, the signal and the LO come from the same laser, but since the AOM shifts the pulse’s frequency, the backscattering will be centered at the frequency difference $\omega_{s}$ − $\omega_{L}$ and can be obtained using analog or digital downconversion. Assuming the difference between the signal’s initial phase and that of the LO are the same, i.e., $\theta_{s}$ − $\theta_{L}$ = 0, the four in-phase and quadrature component outputs are simplified as follows:

(24) $$I_{x}\left(t\right)=\frac{R}{2}A_{sx}\left(t\right)A_{Lx}cos\left(\theta (t\right))$$

(25) $$Q_{x}\left(t\right)=\frac{R}{2}A_{sx}\left(t\right)A_{Lx}sin\left(\theta (t\right))$$

(26) $$I_{y}\left(t\right)=\frac{R}{2}A_{sy}\left(t\right)A_{Ly}cos\left(\theta (t\right))$$

(27) $$Q_{y}\left(t\right)=\frac{R}{2}A_{sy}\left(t\right)A_{Ly}sin\left(\theta (t\right))$$

Then, the amplitude and the phase of the RBS along the fiber can be obtained from the combined in-phase and quadrature components, *I* and *Q*, as

(28) $$A_{s}=\sqrt{{\left(I_{x}\left(t\right)\right)}^{2}+{\left(I_{y}\left(t\right)\right)}^{2}+{\left(Q_{x}\left(t\right)\right)}^{2}+{\left(Q_{y}\left(t\right)\right)}^{2})}$$

(29) $$\theta \left(t\right)=tan^{-1}\left(\frac{Q_{x}\left(t\right)+Q_{y}\left(t\right)}{I_{x}\left(t\right)+I_{y}\left(t\right)}\right)$$

Finally, after phase unwrapping, the phase change induced by any perturbation along the fiber can be demodulated by subtracting the phase from an adjacent position:

(30) $$▵\theta =\theta_{z2}\left(t\right)-\theta_{z1}\left(t\right)$$

Among other signal processing methods in DAS, the FFT is used to obtain the frequency response of vibrations at each sensing point along the fiber, and it is an efficient way of calculating the N-point DFT, which for a discrete signal *X* is given by

$$X\left(k\right)=\sum_{n=0}^{N-1}x\left(n\right)W_{N}^{kn}\;\mathrm{for}\;k=0,1,\ldots ,N-1$$

where

$$W_{N}=e^{-j\frac{2\pi }{N}}\;\mathrm{for}\;N=2,4,8,16,\ldots$$

### 2.2. Simulation of Cloud Computing with CloudSim

The concept of cloud computing describes a computing paradigm where shared resources are used to run applications rather than relying solely on local servers or personal devices. Similar to cloud computing, grid computing leverages the unused processing power of all computers connected to a network to solve complex problems that are beyond the capabilities of a single standalone system [34,35]. Cloud computing is gaining preference due to several factors. Cloud services offer flexibility, exhibit dynamic behavior, operate without the need for dedicated servers, and feature NoSQL databases that provide significantly lower access latency. Examples of cloud storage services include Google Drive, Dropbox, OneDrive, and MediaFire. These advantages have contributed to the widespread use of cloud computing in developing scalable solutions across industries such as retail, finance, transportation, and entertainment, where consistently fast response times are achieved even under peak load conditions involving tens of millions of requests [36].

Cloud infrastructure components, such as data centers, VMs, resource provisioning strategies, and the entire behavior of cloud systems, can be simulated using CloudSim tools. The application provisioning strategies are generic and highly extensible. CloudSim currently has the capability to simulate and model cloud environments, including both individual clouds and interconnected clouds. Researchers have utilized CloudSim for investigating cloud resource provisioning and energy-efficient management of data center resources [37]. The tool, which is primarily developed in Java, is freely available under the LGPL license. A comprehensive discussion regarding cloud computing architectures can be found in [38,39,40].

## 3. Experimental Setup

**Figure 3:**
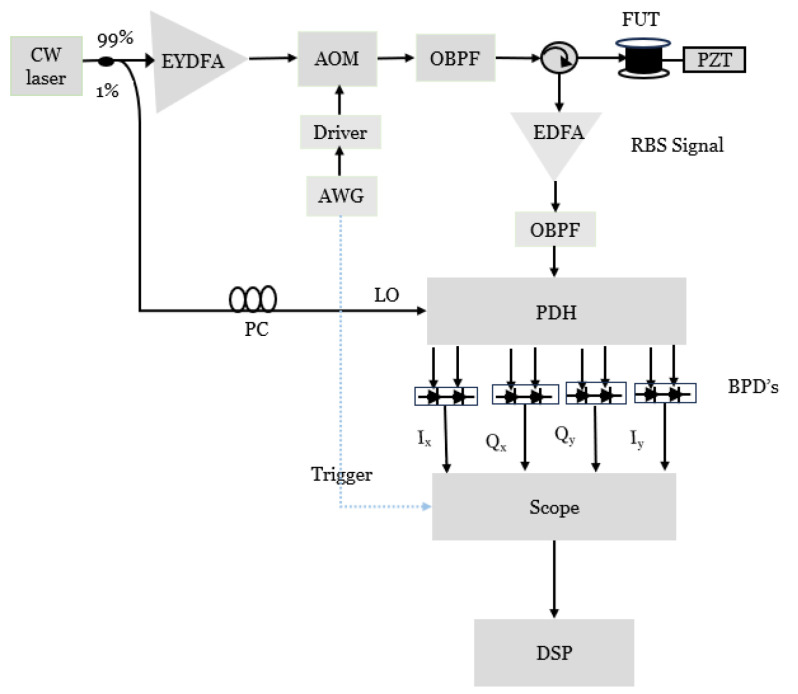
Experimental setup.

The experimental setup of the φ-OTDR based on the polarization diversity hybrid scheme is shown in Figure 3. The continuous light emitted by a narrow linewidth laser source operating at 1550 nm and a linewidth of 100 Hz was split by a 99:1 coupler. Then the light in the upper branch was amplified by an erbium–ytterbium doped fiber amplifier (EYDFA) and injected into an acoustic–optic modulator (AOM) to generate pulses with a width of 100 ns and a repetition rate of 8.33 kHz. Then, an optical bandpass filter was used to remove the amplified spontaneous emission (ASE) noise, and the filtered pulse was launched into a 10 km single-mode sensing fiber through a circulator.

Then, the filtered light was injected into a 90° polarization diversity hybrid (PDH), which consists of two single-polarization 90° optical hybrids for extraction of change in the phase and amplitude induced by perturbations while suppressing polarization fading. Finally, the LO in the lower branch and the backscattering signal were mixed in a 90° optical hybrid. A polarization controller (PC) was inserted in the lower branch to match the polarization of the LO with that of the signal. The output of the PDH was given to the four balanced photodetectors to detect the beating that had a 100 MHz bandwidth.

## 4. Design of a Signal Processing Scheme for Long-Range DAS Using CloudSim

**Figure 4:**
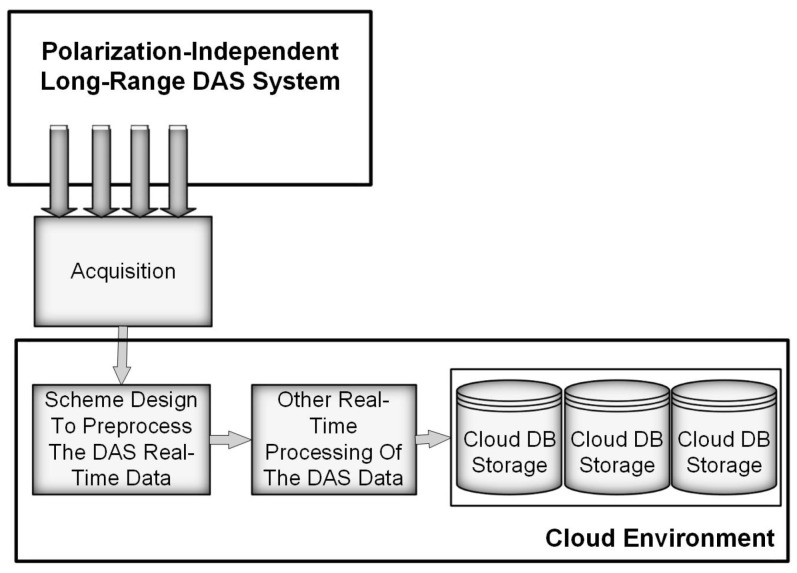
Block diagram of the developed system [25].

Figure 4 shows a system designed to integrate a DAS system with cloud services. In a cloud environment, one of the key components is the system developed for transmitting large-scale data to cloud storage. This requires designing and implementing a method for efficiently transferring the substantial amounts of data generated by the DAS system to the cloud. Another essential element is the framework created for the real-time preprocessing of DAS data, which includes the steps needed to prepare the data for both storage and subsequent analysis [25].

**Figure 5:**
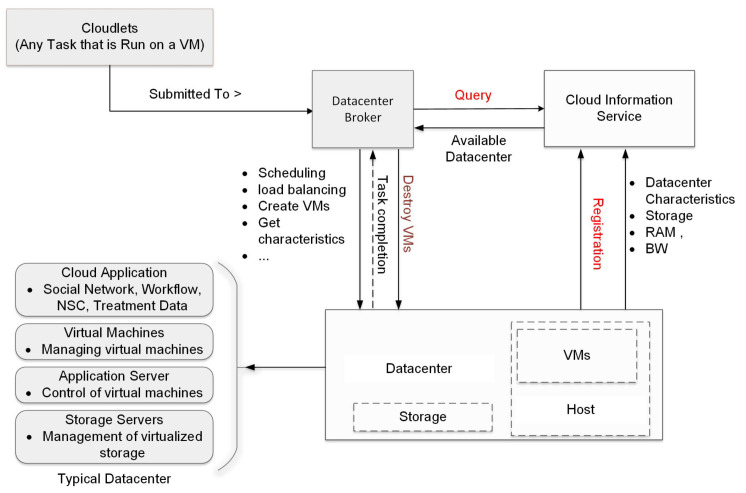
Block diagram of simulation flow for the basic scenario [25].

The step-by-step guide to setting up a cloud simulation project using CloudSim (cloudsim-5.0) can be found in [25]. The simulation flow for the signal processing of sensor data in a CloudSim framework is shown in Figure 5. The simulation starts with initializing CloudSim, creating a data center for managing cloudlet tasks on VMs. A broker then allocates resources and schedules tasks across VMs. After setting up, the simulation runs until tasks finish, followed by a summary of execution times and resource usage to assess cloud performance. The full explanation of the simulation process can be found in [25].

**Figure 6:**
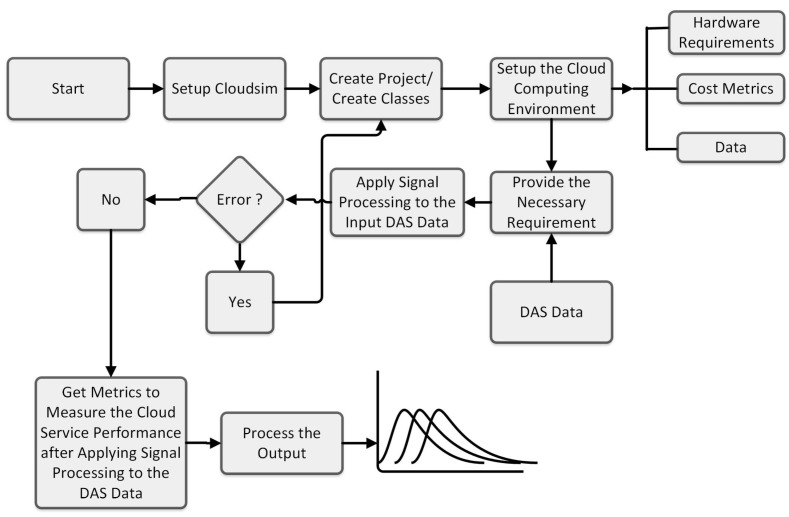
Schematic representation of the implementation of signal processing of DAS sensor data in CloudSim [25].

The schematic in Figure 6 represents implementation of a signal processing system for a DAS sensor using CloudSim. After setting up CloudSim, the next step is understanding its components and functionalities. System requirements, including hardware and data, are then defined, followed by creating project classes for data centers, VMs, and cloudlets. The simulation runs several different Java classes for differential and FFT computations. If errors occur in any stage of the processing, class creation is revisited; otherwise, outputs are generated for further MATLAB analysis, which includes visualization and statistical analysis. The detailed descriptions of the workflow can be found in [25].

## 5. Results and Discussion

**Figure 7:**
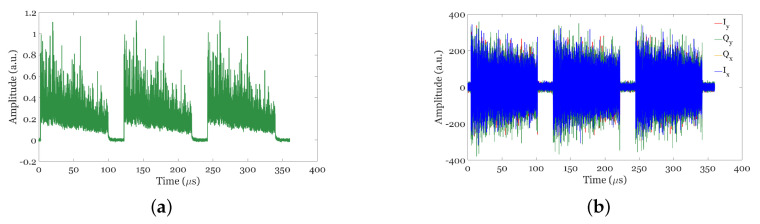
Sample of 3 RBS traces: (**a**) Before being fed to the PDH. (**b**) Overlapped raw traces from the four outputs of the PDH.

Figure 7a shows the coherent Rayleigh backscattering traces before they are fed to the polarization diversity hybrid, and Figure 7b shows the overlaps of the raw $I_{x}$, $Q_{x}$, $I_{y}$, and $Q_{x}$ after the PDH, proving that the RBS traces have high SNR and exhibit common-mode noise suppression.

**Figure 8:**
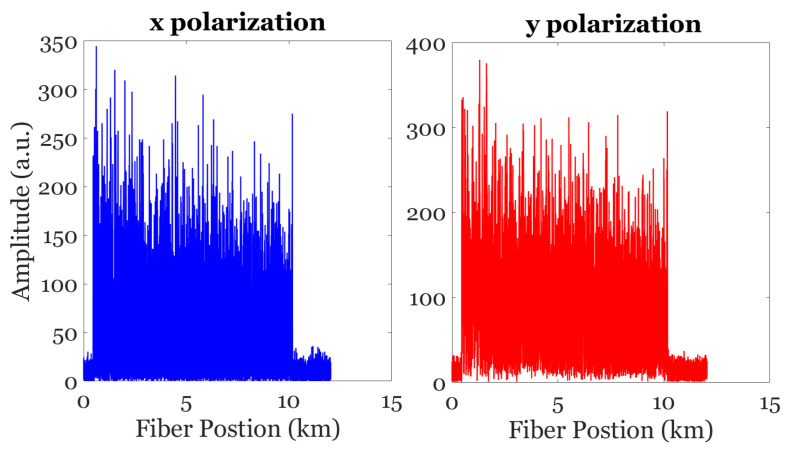
Demodulated amplitude traces. Left: *x* polarization; right: *y* polarization.

The amplitude of the individual polarization components (*x* and *y*) with no averaging are reported in Figure 8. As illustrated in the figure, the signal-to-noise ratios (SNRs) of the two individual amplitude traces differ due to the effects of polarization.

### 5.1. Processing Times for Varying VM Capacity

In the signal processing scheme which is designed and evaluated using the CloudSim framework, cloudlet lengths are carefully measured in Million Instructions (MI) to accurately assess the computational intensity of various operations. Specifically, the processing is applied to the equivalent size of the samples in the sensor data from real-time measurements in polarization-independent long-range DAS using delayed self-mixing of coherent Rayleigh backscattering signals in an 18,750 by 416 matrix, which is a total of 7,812,504 numbers.

With regard to representation formats, single-precision floating-point numbers are used to represent real numbers, following the IEEE 754 standard which requires 4 bytes (32 bits) of storage per number. This means that each individual sample, whether it is an input or an output in a computational process, occupies 4 bytes of memory.

In the designed computing scheme, where the first batch of data contains 781,250 numbers, to calculate the total memory or storage size required for this batch, we multiply the number of samples by the memory size for each value: 781,250 numbers×4bytespernumber= 3,125,000 bytes. Considering 1 MB = 1,048,576 bytes, the size in megabytes is $\frac{3,125,000\;\mathrm{bytes}}{1,048,576}\approx 2.98\;\mathrm{MB}$. Hence, we use 4 MB for the total memory requirements for the first batch. Then, as the batch size increases, the total file size increases proportionally. For instance, if the batch size doubles, the data size also doubles, leading to a corresponding increase in memory usage.

This calculation applies to both the input and the output files if the output data follow the same format. Therefore, each time the batch size increases, the file sizes for both input and output will grow proportionally. In this paper, we are processing batches of increasing size, so the memory or storage demand for both input and output files will increase consistently.

Regarding the computational intensity of all the computations involved in the real-time measurements in a polarization-independent long-range DAS, the first step in the proposed system is the polarization diversity computation, which serves as a crucial preprocessing stage for data analysis. In this phase, we process a total of 10 batches of data. To enhance real-time processing capabilities, we plan to generate an additional 10 batches within each of the initial batches, resulting in a total of 100 batches to effectively manage the data flow.

Each batch comprises approximately 781,250 samples. The signal processing for polarization-independent measurements in DAS involves various mathematical operations, including additions, multiplications, and divisions. To assess the computational intensity of these operations, we assume that the data will first be converted into binary representation before being processed by computer circuits.

The operations involve substantial computations, whose load is evaluated by expressing it in terms of MI. After conducting the necessary calculations for the first batch, we estimate that the total computational requirement amounts to approximately 487.5 MI.

As we progress through the subsequent batches, we anticipate an increase in the number of instructions required. This escalation in computational intensity can be attributed to various factors, including the cumulative data from previous batches and the complexity of the operations involved in the polarization diversity algorithm. Each following batch will therefore require an increasingly larger number of MI, reflecting the growing computational demands of processing larger datasets and executing more complex operations.

**Figure 9:**
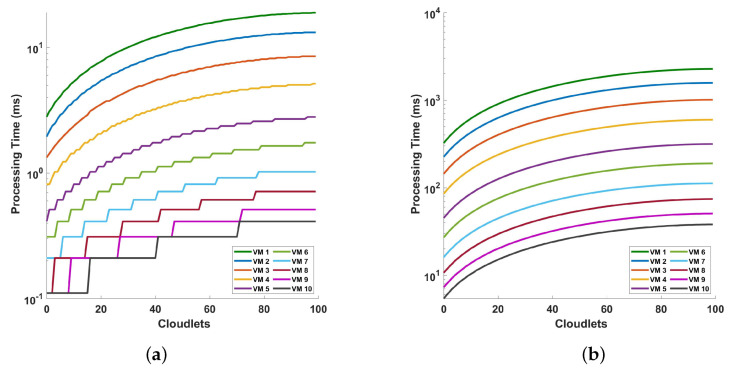
Analysis of processing time and cloudlet utilization for the preprocessing focusing on two distinct scenarios comprising the following: (**a**) 416 consecutive cycles of measurements where 18,750 samples are taken for a single cycle measurement, and (**b**) 832 consecutive cycles of measurement where 468,750 samples are taken for a single cycle measurement. Note that the number of cloudlets increases for each cloudlet ID on the horizontal axis. The measurements are conducted in a 10 km optical fiber.

Figure 9 shows the relationship between the processing time and cloudlet length for preprocessing operations. In plot (a), the processing time for VM 1 when processing 100 cloudlets is approximately 18.94450 ms, while for the best-performing VM 10, the processing time is 0.41222 ms. This indicates a substantial difference in performance between the worst and best VMs in this scenario. In contrast, in plot (b), the processing time for VM 1 for the same number of cloudlets (100) is about 51,891.42998 ms, and for VM 10, it is 38.13402 ms. The plots show not only that as the sample size per cycle increases, the computational intensity of the preprocessing increases but also that as the VM capacity increases, the variability in processing times gets discretized, as can be seen in the staircase-type plots starting from VM 5.

The computations involved in calculating the differential based on magnitude are quite complex and consist of several mathematical operations. These operations include additions, multiplications, and a series of further additions, subtractions, and multiplications, particularly for the square root calculation. The square root function, in many computational algorithms, involves an iterative process where multiple arithmetic operations are carried out to approximate the result. This makes the magnitude computation relatively intensive when processing large batches of data. In our system, we previously assumed batch processing to optimize performance and handle real-time requirements. As stated earlier, for the first batch, we are limiting the number of samples per trace in order to streamline the processing. Specifically, we are working with 1875 samples per trace and a total of 416 traces for the first batch.

For this first batch of data, after performing the necessary calculations, we estimate that the total number of instructions required for the magnitude computation is approximately 1248 MI. This estimation includes all the arithmetic operations mentioned earlier, including the square root operations, which are computationally expensive due to the iterative steps involved in achieving an accurate result. The value of 1248 MI is based on the size of the batch (1875 samples per trace and 416 traces) and the complexity of the operations performed during the magnitude calculation. As we process additional batches, we will compute the required number of instructions for each batch in a similar manner. The computational intensity is expected to increase as we process larger batches or apply more complex algorithms. Furthermore, for each batch, we will generate another 10 sub-batches to ensure that the system maintains real-time processing capabilities. These sub-batches allow us to handle data incrementally, improving the efficiency and speed of the overall system without overwhelming the computational resources.

Once we have calculated the instructions required for the magnitude computation, the next step is to calculate the instructions needed for the differential operation. The differential operation, while less computationally intensive than the magnitude calculation, still involves a series of arithmetic operations, primarily additions and subtractions. For the first batch, we estimate that the total number of instructions required for the differential operation is is calculated to be 20 MI. This relatively lower number reflects the fact that the differential operation is simpler and requires fewer complex computations compared to the magnitude calculation. By combining the computational loads from both the magnitude and differential operations, we gain a clearer understanding of the total processing requirements for the first batch. As we move forward with the subsequent batches, we will repeat this process, adjusting the instruction counts as needed based on the size and complexity of each batch. The goal is to ensure that our system can handle the data processing efficiently while maintaining the accuracy of the computations.

**Figure 10:**
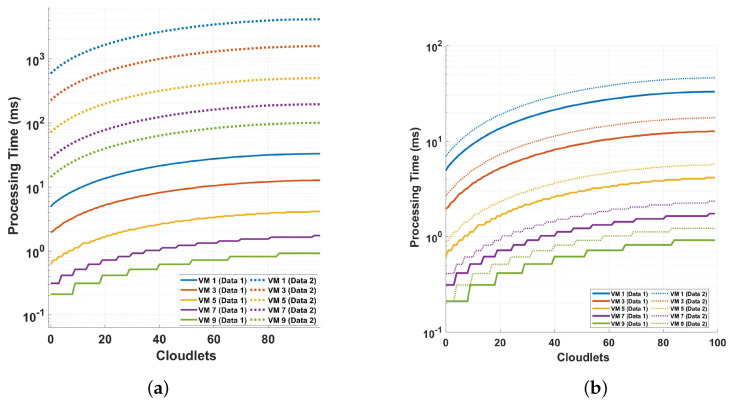
Processing time and cloudlet utilization for the differential operation of the system when using the magnitude value for the detection for different cycles of measurements performed varying the samples per cycle: (**a**) comparison of two different sampling schemes discussed in the previous figure with solid lines indicated as Data 1 for 18,750 samples and broken lines for 468,750 samples indicated as Data 2, both for magnitude differential operation, and (**b**) comparing the differential operation without the preprocessing shown as Data 1, and with preprocessing, shown as Data 2.

Figure 10 shows an investigation of the processing time and cloudlet utilization in the differential operation of a polarization-independent long-range DAS system, focusing on the detection using the magnitude value. The measurements without preprocessing are labeled as *Data 1*, while those that involve preprocessing are shown as *Data 2*. Two distinct scenarios are examined to understand the impact of different measurement cycles and preprocessing methods on the system’s performance. In plot (a), the solid line (Data 1) represents the processing time for VM 1 at 100 cloudlets, where the maximum big data processing time is approximately 47.59345 ms. For VM 9, the processing time is significantly lower, at around 0.92227 ms. The broken line (Data 2) in plot (a) shows that for VM 1, the processing time for 100 cloudlets is about 6001.84080 ms, while for VM 9, it is around 133.55787 ms. The second scenario (plot b) focuses on the differential operation of the system, comparing the results obtained with and without preprocessing. This comparison aims to assess the impact of preprocessing on the system’s detection capabilities and overall performance. Through these two scenarios, the analysis offers valuable insights into the influence of different measurement configurations and preprocessing on the system’s processing time and cloudlet utilization, thus contributing to a better understanding of the DAS system’s performance under varying conditions. In plot (b), the solid line again shows the processing time for VM 1 at 100 cloudlets to be about 47.59345 ms, the same as in plot (a) for Data 1, because it is the same process. For VM 9, the processing time is 1.22535 ms. For the broken line in plot (b), The processing time for VM 1 at 100 cloudlets is about 66.29941 ms. It can also be seen that VM 9’s processing time for 100 cloudlets is 1.63002 ms. When comparing the two plots, the solid lines (Data 1) in both plot (a) and plot (b) are identical, because it is the same process. However, the broken lines (Data 2) reveal significant differences. As shown in plot (a), the processing times for VM 1 and VM 9 are much higher in Data 2, with VM 1 taking about 6001.84080 ms and VM 9 taking around 133.55787 ms. Comparing plot (a) and plot (b), considering the solid lines and broken lines, shows that the preprocessing does not add a significant computation load compared to adding more columns to the data (i.e., adding more samples per cycle). This shows that the preprocessing added to make the DAS system polarization-independent is not computationally intensive.

**Figure 11:**
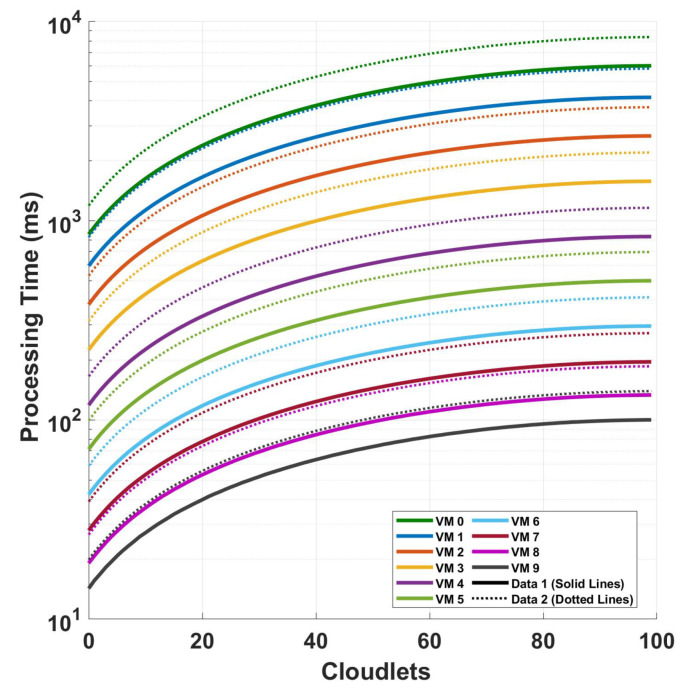
Processing time and cloudlet utilization for the differential operation on the DAS data in the cloud environment when using the magnitude value for the detection, showing a comparison of the effect of adding the preprocessing (polarization diversity computation) to our computation. The analysis focuses on the two distinct scenarios described in Figure 10.

Figure 11 shows the analysis of processing time and cloudlet utilization for the differential operation of a polarization-independent long-range DAS system, where the detection is based on the magnitude value. The primary focus is to assess the effect of introducing preprocessing for polarization diversity computation. In the plot, the solid line (Data 1) indicates that the processing time for VM 1 when handling 100 cloudlets (the maximum data size) is approximately 6001.84080 ms. For VM 9, the processing time for the same workload is about 100.15572 ms. The broken line (Data 2) in the plot further accentuates this difference. For VM 1, the processing time for 100 cloudlets under the conditions of Data 2 is even higher, reaching around 8365.61552 ms. Similarly, for VM 9, the processing time also increases, reaching approximately 139.55369 ms. This shows that the conditions represented by Data 2 lead to generally higher processing times for all VMs. Similar to the case of differential operations, the results indicate that the preprocessing step does not significantly increase computational load compared to adding more columns to the data (i.e., increasing the number of samples per cycle), once again confirming that the preprocessing applied to achieve polarization independence in the DAS system is not computationally intensive. The process of calculating the differential traces from phase involves a computationally intensive algorithm for phase calculations from in-phase and quadrature components with subsequent phase unwrapping and, finally, the computation of the differential phase itself, which is need for calculating the phase change relative to an adjacent point.

One of the most computationally demanding aspects of phase data processing is the phase unwrapping operation. Due to the nature of phase measurements, phase data often appear wrapped, meaning that they are restricted to a certain range (typically between −π/2 and π/2). To increase the range, we need to “unwrap” the phase, effectively reconstructing the true phase values by removing discontinuities. Phase unwrapping algorithms are iterative in nature and involve complex operations, often requiring a significant amount of computational resources to ensure accuracy and avoid errors, particularly when dealing with large datasets.

The unwrapping operation is aimed at extending the range of the arctan function, and its most common implementation consists of the following steps: For each value of the phase θ(i) for i[1,N],N being the total number samples, it iteratively calculates the difference between two adjacent values $\delta_{i}=\theta \left(i\right)-\theta \left(i-1\right)$. If $\delta_{i}>\pi$, it subtracts 2π from all values of θ(k) in the range [i,N]. If instead $\delta_{i}<-\theta$, it adds 2π to all subsequent values of θ(k) in the range [i,N] [41].

Finally, the differential operation itself is performed after the previous stages. The differential operation involves calculating the change in the unwrapped phase with respect to an adjacent point. While this step is less complex than phase unwrapping, for distributed sensing, it still involves a series of addition and subtraction operations over a large number of samples, contributing to the overall computational load. Based on the batch processing system assumed earlier, for the first batch, we have a data structure that includes 1875 samples per trace and a total of 416 traces.

After accounting for all the steps in the phase differential computation—preprocessing, phase calculation, phase unwrapping, and the differential operation—the total computational load for the first batch is estimated to be approximately 2624 MI. This figure represents the cumulative computational intensity of all the aforementioned processes required to process the phase data in a single batch. As we proceed to process additional batches, the MI values for each subsequent batch will be calculated accordingly, following the same approach as outlined for the first batch. Importantly, as previously discussed, we will also generate 10 sub-batches within each batch to enhance the system’s ability to perform real-time processing. This subdivision of batches ensures that the system can handle incremental processing and improve overall efficiency, allowing for more manageable and scalable computational loads as the data are processed.

**Figure 12:**
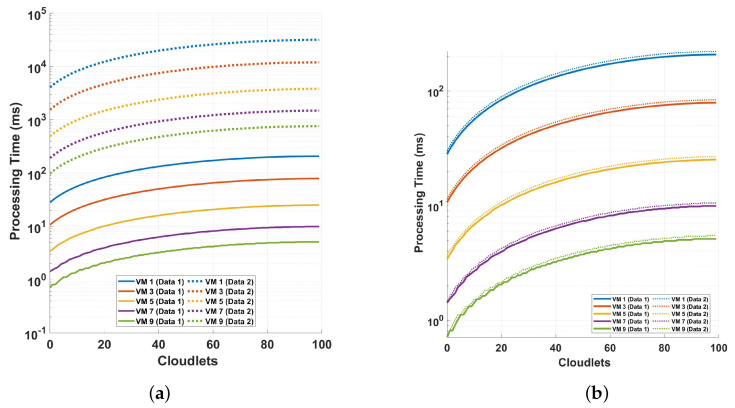
Processing time and cloudlet utilization for the FFT operation when using the magnitude value for the detection. An analysis on different cycles of measurements with varying the samples-per-cycle measurement points. The analysis focuses on two distinct scenarios as stated in the previous figure. It is the same except that this is for the FFT operation.

Figure 12 shows the relationship between the processing time and cloudlet length for the magnitude FFT operation. The details for the figure are the same as in Figure 10 except that this is for FFT. In plot (a), the solid line (Data 1) shows that the processing time for VM 1 when handling 100 cloudlets (the maximum big data) is approximately 299.66082 ms. For VM 9, the processing time under the same conditions is much lower, at around 6.83191 ms. The broken line (Data 2) in plot (a) shows that VM 1’s processing time for 100 cloudlets is dramatically higher, around 45,692.94081 ms, while VM 9 processes the same workload in about 1015.41807 ms. In contrast, in plot (b), the solid line represents the same processing times as in plot (a), where VM 1 takes about 299.66082 ms and VM 9 takes 5.13126 ms for processing 100 cloudlets (the maximum big data). However, the broken line in plot (b) presents less processing time compared to plot (a). In plot (b), VM 1’s processing time for 100 cloudlets (the maximum data) is about 318.37834 ms, which is significantly lower than the 45,692.94081 ms seen in plot (a). Similarly, VM 9’s processing time is only 7.20945 ms, slightly higher than the time seen in the solid line but still far lower than the 1015.41807 ms in plot (a) under Data 2 conditions. The comparison between plot (a) and plot (b) reveals what we have already observed in previous results. We can also see that the FFT computation needs more resources than the differential operation. From the plots, we can see that it requires almost five times the computations required for the differential.

The computation of the FFT is a crucial step in many signal processing applications, and it is computationally intensive due to the large number of arithmetic operations required, particularly when dealing with large datasets. For the first batch of data, we calculate the computational intensity of the magnitude FFT to be approximately 5863 MI. This value is based on the size of the batch and the complexity of the FFT algorithm. In addition to this, we must also account for the computational requirements of the magnitude calculation itself, which we previously determined to be around 1245 MI. Therefore, for the first batch, the total computational intensity for both the magnitude FFT and magnitude calculation is the sum of these two values, yielding a total of approximately 7108 MI.

With regard to the FFT of the phase, for the first batch, the computational intensity is expected to be similar to that of the magnitude FFT, which is around 5863 MI. However, the phase FFT also requires additional computations for the phase unwrapping algorithm. These steps are necessary to prepare the phase data for FFT analysis and, as noted earlier, are computationally demanding. The phase sensitivity calculation and phase unwrapping together add a significant number of instructions to the overall computational load. After adding these requirements, we estimate that the total computational intensity for the phase FFT and associated operations for the first batch comes to approximately 7968 MI. Finally, we need to add the computational intensity requirement of the preprocessing, which will result in total computational intensity of approximately 8468 MI.

As with our previous calculations, we will extend this approach to subsequent batches, ensuring that the computational intensity is evaluated in batches. Each additional batch will be processed similarly, and the total computational requirements will increase proportionally as the size of the data grows. Additionally, the earlier consideration of processing 10 sub-batches per batch will apply here as well, ensuring that the system maintains real-time processing capabilities while handling large datasets efficiently.

**Figure 13:**
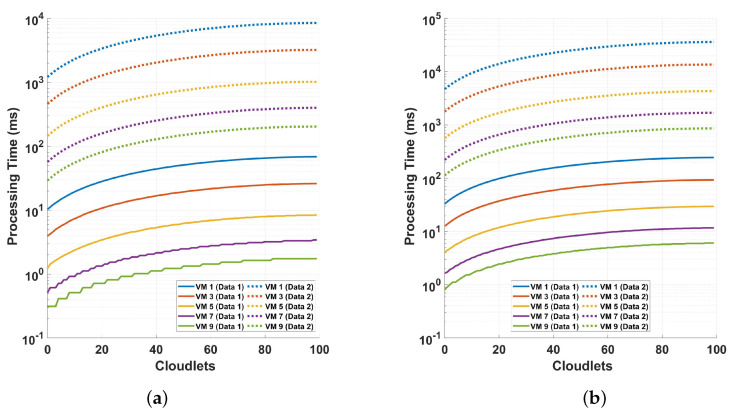
Examination of processing time and cloudlet utilization for the phase differential and phase FFT operation: an analysis on different cycles of measurements with varying the samples-per-cycle measurement points. The analysis focuses on two distinct scenarios: (**a**) comparing two different sampling sizes discussed in previous analyses (solid lines indicated as Data 1 for 18,750 samples and broken lines for 468,750 samples indicated as Data 2) for phase differential computation, and (**b**) the same analysis as in (**a**) but for phase FFT processing.

Figure 13 depicts the processing time and cloudlet utilization, focusing on phase differential and phase FFT operations. The analysis is based on measurements conducted over different cycles, with varying numbers of samples per cycle, to evaluate the system’s performance under two distinct scenarios. In the first scenario (plot a), the system’s performance is analyzed using phase differential computation. In plot (a), the solid line (Data 1) shows that the processing time for VM 1 at 100 cloudlets is approximately 98.36686 ms, while for VM 9, the processing time is much shorter, around 2.37368 ms. This highlights a significant performance gap between the least and most efficient VMs in handling large data. The broken line (Data 2) in plot (a) indicates a substantial increase in processing time for both VMs under different data conditions. For VM 1, the processing time for 100 cloudlets rises to around 12,227.41954 ms, while for VM 9, it increases to about 271.90426 ms. The second scenario replicates the same conditions as the first but focuses on phase FFT processing instead of phase differential computation. In both scenarios, the results offer a detailed understanding of how different computational approaches—phase differential and phase FFT—affect the overall performance of the DAS system when applied to long-range optical sensing over multiple cycles and varying sample sizes. In plot (b), the solid line shows the processing time for VM 1 at 100 cloudlets as approximately 352.31586 ms, which is significantly higher compared to plot (a). Similarly, VM 9 processes the same workload in about 8.01542 ms which, while still faster than VM 1, is much higher than its corresponding time in plot (a). The broken line in plot (b) depicts an even larger processing time for both VMs when handling the maximum data. VM 1’s processing time reaches around 51,891.42998 ms, while VM 9’s processing time increases to 1153.14877 ms, further illustrating the increase in workload. Comparing the two plots, it is evident that the FFT operation is more computationally intense than the differential operation while using the phase for the computation in both cases. The analysis also shows that the phase computation is more intense that the magnitude computation. Note that the phase calculation includes the arctan and the phase unwrapping computations.

**Figure 14:**
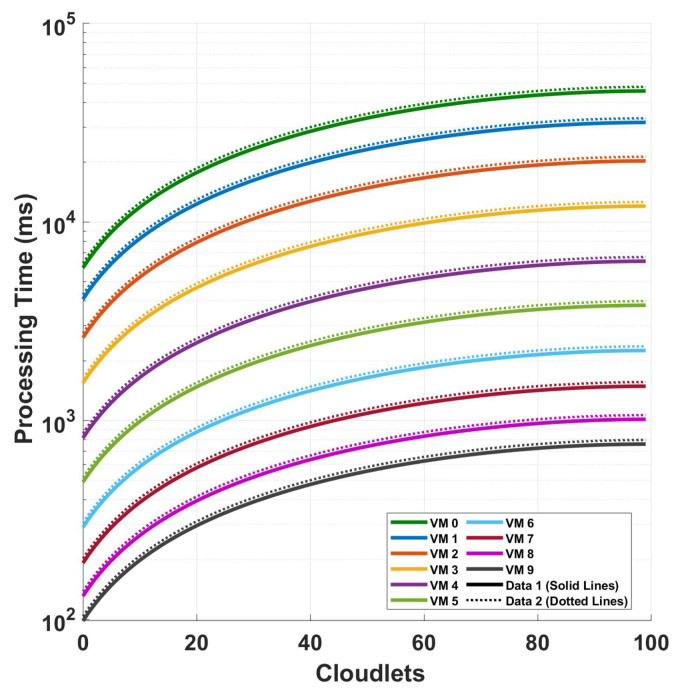
Investigation of processing time and cloudlet utilization for the FFT operation when using the magnitude value for the detection to compare the effect of adding the preprocessing (polarization diversity computation) to our computation. The analysis focuses on two different sampling scenarios discussed in the previous figures.

Figure 14 shows the relationship between the processing time and cloudlet length for the magnitude FFT operation when using the magnitude value for the detection including the effect of the computational intensity of the preprocessing. This relationship is examined under two distinct scenarios, a 416 cycle of measurement and 832 cycles of measurement, both conducted for a polarization-independent long-range DAS system. In the plot, the solid line shows that the processing time for VM 1 at 100 cloudlets (the maximum data size) is approximately 45,692.94081 ms. For the best-performing VM, VM 9, the processing time for the same workload is significantly lower, at around 761.59642 ms. The broken line in the plot illustrates a similar trend, with processing times increasing slightly for both VMs. The processing time for the VM for 100 cloudlets is approximately 47,942.07757 ms, which is higher than the time seen in Data 1. Similarly, VM 9’s processing time increases slightly to around 799.03595 ms. A comparison of the plots shows that as the computational intensity of the system increases either by increasing the cycles or by increasing the samples per cycle, the preprocessing computational increment on all of the system’s computational intensity becomes smaller and smaller.

### 5.2. Mean Processing Times for Varying VM Capacity and Incremental Sample Sizes

**Figure 15:**
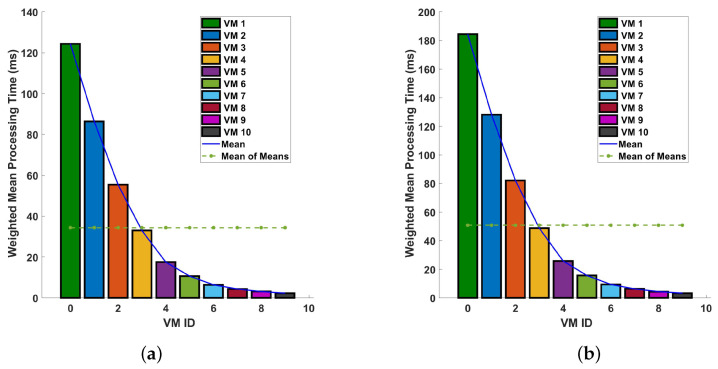
Determining the mean processing time for each virtual machine in differential operations: a comparative analysis on a single cycle versus multiple cycles in a 10 km optical fiber. The investigation is conducted under two distinct conditions: (**a**) the magnitude differential operation with the preprocessing included, and (**b**) the phase differential operation with the preprocessing included.

In Figure 15, we analyze the mean processing time for each virtual machine in differential operations. In comparing the two plots, in plot (a), the maximum mean execution time is around 120 ms, while the minimum is approximately 5 ms. This results in a range of 115 ms between the highest and lowest execution times. On the other hand, in plot (b), the maximum mean execution time reaches 180 ms, and the minimum mean execution time is about 5 ms, giving a wider range of 175 ms. This shows that the processing time differences between with and without the preprocessing becomes insignificant when an efficient VM is used.

**Figure 16:**
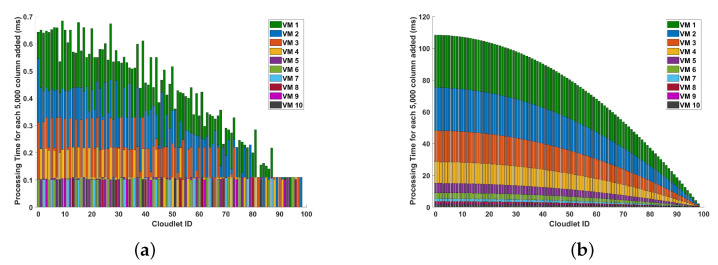
Change in processing time for incremental data in optical fiber measurements (for each additional column) during the magnitude differential operations: (**a**) for every increment of approximately 200 columns, and (**b**) for every increment of approximately 5000 columns. The measurements are conducted in a 10 km long optical fiber. This examination aims to understand the computational scalability of these operations in the context of increasing data volume.

Figure 16 depicts the processing time associated with incremental data in optical fiber measurements during magnitude differential operations. The focus is on examining how the system’s computational performance scales as the data volume increases, particularly in the context of long-range optical sensing. In comparing the two plots, we observe significant differences in how the increase in processing time behaves as the number of columns increases. In plot (a), the maximum increment in execution time for every 200 columns is approximately 0.64288 ms, while the minimum increment is negligible. This suggests that for smaller computational loads, such as 200 columns, the execution time increases in relatively small increments. In plot (b), the maximum increment in execution time for every 5000 columns is substantially larger, reaching 108.396 ms, while the minimum increment is still negligible. We can see from the plots that as the amount of additional samples per cycle increases, the increment in processing time for each added batch decreases. It is also evident that as the amount of additional samples per cycle increases, the increment in processing time for each added batch diminishes regardless of the VM picked, and this decrease is observed when adding batch sizes of both 200 and 5000 columns, hence confirming its scalability.

## 6. Conclusions

The plots for the preprocessing time illustrate that the added preprocessing steps to achieve polarization independence in the DAS system do not introduce a substantial computational overhead. This highlights that while other operations contribute to the overall computational intensity, the specific preprocessing required to make the system polarization-independent remains relatively lightweight in comparison.

Our analysis on the computational times also suggest that as the system’s computational load increases—whether by adding more cycles or increasing the number of samples per cycle—the relative impact of the preprocessing on the system’s total computational intensity diminishes. In other words, the preprocessing becomes less of a bottleneck as the overall system’s computational requirements grow. This observation is reinforced by the mean execution time plot, which shows that when using a highly efficient VM, the difference in processing time between scenarios with and without preprocessing becomes almost negligible. The preprocessing step, while necessary for making the DAS system polarization-independent, does not significantly hinder system performance in such configurations.

Furthermore, the incremental plots highlight a key aspect of scalability: as the number of samples per cycle increases, the additional processing time required for each successive batch of sample increments diminishes. This demonstrates that the system is capable of handling increased data throughput efficiently, as the system’s performance scales well with the growing input size. The scalability is maintained even when the system is made polarization-independent, ensuring the processing in the dynamic sensing system can manage larger datasets without encountering detrimental computational slowdown.

In summary, the findings emphasize that although certain operations, such as FFT and phase-based processing, impose higher computational demands, the preprocessing added to achieve polarization independence remains computationally manageable. Moreover, the system exhibits strong scalability, where increasing the number of samples per cycle leads to diminishing processing time increments, further supporting its capacity to handle large-scale operations.

## Data Availability

Data are contained within the article.

## Abbreviations

The following abbreviations are used in this manuscript:

ADC	Analog-to-digital Converter
AOM	Acoustic–optic Modulator
ASE	Amplified spontaneous emission
BPD	Balance Photodetector
DAS	Distributed Acoustic Sensing
DFOIS	Distributed Fiber-Optic Intrusion Sensor
DSP	Digital Signal Processing
EYDFA	Erbium-ytterbium doped fiber amplifier
FFT	Fast Fourier Transfer
FUT	Fiber Under Test
LO	Local oscillator
LD	Linear Dichroism
MIPS	Million Instruction Per Second
ML	Machine learning
NoSQL	Not only SQL
PBDs	Pair of balanced photodetectors
PBS	Polarization beam splitter
PC	Polarization controller
PDH	Polarization diversity hybrid
PRS	Pattern Recognition Systems
PZT	Piezoelectric Transducer
RBS	Rayleigh backscattering
SMF	Single-mode fiber
SNR	Signal-to-noise ratio
SOP	State of polarization
VM	Virtual machine
φ-OTDR	Phase-Sensitive Optical Time Domain Reflectometery
